# Small extracellular vesicles as system-level regulators and predictive biomarkers in breast cancer progression and chemoresistance

**DOI:** 10.3389/fphar.2026.1804877

**Published:** 2026-03-20

**Authors:** Jiaxiu Li, Hengzheng Cheng, Xiao Liu

**Affiliations:** Department of General Surgery, The Affiliated Wuxi People’s Hospital of Nanjing Medical University, Wuxi, China

**Keywords:** breast cancer, chemotherapy resistance, predictive biomarkers, regulatory networks, small extracellular vesicles, tumor microenvironment, tumor progression

## Abstract

Small extracellular vesicles (sEVs) play a pivotal regulatory role in intercellular communication within breast cancer, coordinating tumor growth, microenvironment remodeling, immune modulation, and therapeutic response. Beyond serving as passive carriers of biomolecules, mounting evidence indicates that sEVs actively orchestrate the dynamic evolution of the tumor ecosystem through cargo specificity and cell-of-origin-dependent mechanisms. We synthesize recent research advances (2021–2025) to elucidate how nucleic acids, proteins, and lipids carried by sEVs integrate into key processes in breast cancer, including oncogenic signaling, epithelial-mesenchymal transition (EMT), metastasis microenvironment formation, immune evasion, and therapeutic resistance. We further highlight how their specificity and cellular origin provide a unified framework for reconciling reported functional heterogeneity in sEVs across studies. Building on this, we discuss emerging evidence demonstrating sEVs' role in reshaping tumor-immune and therapeutic ecosystems, promoting immune evasion, and facilitating horizontal transmission of drug-resistant phenotypes. Finally, we evaluate the translational potential of sEVs as biomarkers and therapeutic targets, proposing a conceptual framework for integrating sEV profiling into precision oncology strategies. This review positions sEVs as central regulators of breast cancer ecosystem dynamics, highlighting their potential in reshaping biomarker development and therapeutic innovation within precision oncology.

## 1 Introduction

Breast cancer is the most common malignant tumor among women worldwide. Data indicates that in 2022, its incidence represented 23.8% of all female malignancies, with a mortality rate of 15.4% (Bray et al., 2024). In recent years, although the emergence of new diagnostic and therapeutic technologies has led to a decline in breast cancer mortality (Cardoso et al., 2025), the disease burden remains substantial. The incidence is showing a trend toward younger age groups and continues to be one of the leading causes of cancer-related deaths among women (Bray et al., 2024; Tanne, 2024). Epidemiological studies reveal significant geographical disparities in breast cancer incidence and mortality rates. Middle- and low-income countries exhibit relatively lower incidence but higher mortality rates, likely closely linked to differences in screening coverage and access to treatment resources (Liu C. et al., 2024). Currently, tissue biopsy remains the primary method for clinical diagnosis (Boissière-Michot et al., 2024), staging, and prognosis assessment (Simancas-Racines et al., 2025). However, its inherent limitations—including invasiveness, sampling constraints, and difficulty in dynamic monitoring (Simancas-Racines et al., 2025)—restrict its application in early detection and real-time disease tracking.

The initiation and progression of breast cancer are highly dependent on the tumor microenvironment (TME) (Tan W. et al., 2021), a dynamically regulated network comprising cancer-associated fibroblasts, immune cells, extracellular matrix, and diverse signaling molecules (Yilihamu and Aierxiding, 2025). Within the complex intercellular communications of the TME, sEVs—lipid bilayer nanoparticles measuring approximately 50–150 nm in diameter—exhibit significant biological functions and clinical translational potential due to their high stability, source specificity, concentrated content of information, and ease of acquisition from bodily fluids (Hinger et al., 2020). Secreted by tumor epithelial cells, cancer-associated fibroblasts, and other cell types, sEVs participate in regulating immune responses, intercellular signaling, and microenvironment homeostasis by delivering active molecules such as proteins and nucleic acids, playing a central role in breast cancer progression (Yates et al., 2022a; Yates et al., 2022b).

To overcome the limitations of tissue biopsy, liquid biopsy technology has rapidly advanced. By detecting circulating tumor cells (CTCs), circulating tumor DNA (ctDNA), and extracellular vesicles (EVs) in bodily fluids like blood, this technology offers a new pathway for non-invasive, dynamic tumor monitoring (Reduzzi et al., 2024; Lu et al., 2025; Ochiya et al., 2025). However, CTCs and ctDNA still face significant challenges in practical application: CTCs exhibit poor stability in blood, with unclear release mechanisms and difficult source tracing (Cortés-Hernández et al., 2024); while ctDNA exhibits similar limitations. In contrast, sEVs form via endocytosis, carrying highly selective molecular cargo. Their lipid bilayer structure effectively protects contents from degradation, resulting in higher concentrations and greater stability in bodily fluids. Consequently, sEVs more sensitively and specifically reflect the physiological and pathological states of their source cells (Pham et al., 2025). Studies indicate significant differences in the quantity, composition, and biological activity of sEVs between breast cancer patients and healthy individuals. Furthermore, sEVs participate in regulating multiple malignant processes, including tumor proliferation, invasion and metastasis, angiogenesis, and treatment resistance, suggesting their significant application potential as tumor information carriers in breast cancer diagnosis and treatment (Vinik et al., 2020). Emerging evidence indicates that sEVs are not merely byproducts of tumor cells but active regulators of breast cancer progression and therapeutic resistance (Cao et al., 2021). While sEVs are reshaping our understanding of breast cancer progression, resistance, and systemic regulation, this field remains fragmented, particularly in terms of mechanistic integration and translational relevance.

From functional exploration and mechanism refinement to technological innovation and clinical translation, sEVs have become a paradigm for advancing breast cancer research from fundamental biology to clinical application. To organize this complex, fragmented information, we searched the PubMed and Web of Science Core Collection databases for articles published between 2021 and 2025 using the keywords “breast cancer” and “ small extracellular vesicle”. This review aims to summarize the key mechanisms of sEVs in breast cancer development and progression, with a focus on exploring their translational potential in diagnosis, treatment, and prognostic assessment. It seeks to provide new perspectives and a research foundation for precision medicine in breast cancer. This narrative review offers three contributions: (1) analyzing the critical biological functions of sEVs in breast cancer progression; (2) It clarifies the intrinsic relationship between their cargo specificity and cellular origins, along with associated signaling pathways; (3) Identifying novel technologies and targets for sEV-based liquid biopsy biomarkers in early breast cancer diagnosis, treatment efficacy monitoring, and drug resistance prediction. This in-depth analysis of sEVs not only aids in elucidating breast cancer heterogeneity and metastasis mechanisms while identifying key targets for early intervention, but also provides crucial scientific evidence for overcoming treatment resistance and achieving personalized precision therapy.

## 2 Methods

### 2.1 Databases and time range

This review employs a narrative synthesis approach, focusing on the functions, mechanisms, and translational roles of sEVs in breast cancer. Specifically, it systematically reviews and integrates the biological functions of sEVs in breast cancer initiation and progression, the intrinsic relationships between sEV cargo specificity and cellular origins, relevant signaling pathways, and their translational potential in diagnosis, treatment, and prognostic assessment. Literature searches were conducted in the PubMed database and Web of Science Core Collection database, covering the time period from 2021 to 2025.

### 2.2 Search term combinations

PubMed searches combined Medical Subject Headings (MeSH) terms with free-text keywords, including “breast cancer” and “small extracellular vesicles”. For the Web of Science Core Collection, the same keywords were used for subject searches. All searches were restricted to publications within the specified timeframe.

Search fields included: “((“breast neoplasms”[MeSH Terms] OR (“breast”[All Fields] AND “neoplasms”[All Fields]) OR “breast neoplasms”[All Fields] OR (“breast”[All Fields] AND “cancer”[All Fields]) OR “breast cancer” [All Fields]) AND ((“small”[Journal] OR “small”[All Fields]) AND (“extracellular vesicles”[MeSH Terms] OR (“extracellular”[All Fields] AND “vesicles”[All Fields]) OR “extracellular vesicles”[All Fields] OR (“extracellular”[All Fields] AND “vesicle” [All Fields]) OR “extracellular vesicle”[All Fields]))) AND (2021:2025[pdat]); breast cancer (Topic) AND small extracellular vesicle (All Fields) and 2025 or 2024 or 2023 or 2022 or 2021 (Publication Years).

### 2.3 Literature inclusion criteria

Inclusion criteria: (1) Studies investigating the biological functions of sEVs in breast cancer or relevant experimental tumor models; (2) Studies elucidating intrinsic associations between sEV cargo specificity and cellular origins in breast cancer, along with related signaling pathways; (3) Studies reporting novel technologies based on sEVs for liquid biopsy in breast cancer; (4) Original or translational research published in peer-reviewed journals. (5) Exclusion of literature lacking mechanistic evidence. Given the high heterogeneity in experimental models and study designs among included studies, this review did not perform a quantitative meta-analysis but was reported using the Systematically Assessing Narrative Review Articles (SANRA) framework (Baethge et al., 2019).

## 3 Results

### 3.1 Biological functions of sEVs in breast cancer

**FIGURE 1:**
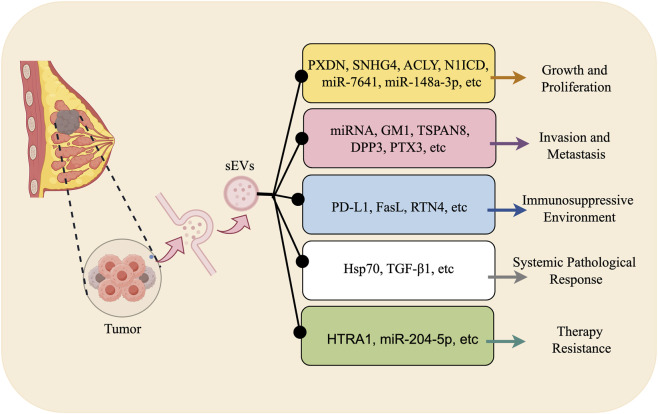
Biological Functions of sEVs in Breast Cancer (By Figdraw). Multifaceted biological functions of small extracellular vesicles in breast cancer. (1) Growth and proliferation: Small extracellular vesicles transport oncogenic molecules such as PXDN, SNHG4, ACLY, and N1ICD, collectively driving tumor cell proliferation and survival. (2) Invasion and metastasis: Small extracellular vesicles deliver miRNAs, GM1, TSPAN8, DPP3, and PTX3, promoting epithelial-mesenchymal transition and tumor invasion/metastasis. (3) Immunosuppressive microenvironment: Small extracellular vesicles display PD-L1, FasL, and RTN4 on their surface, synergistically inhibiting anti-tumor immune responses. (4) Systemic pathological response: Small extracellular vesicles carry Hsp70 and TGF-β1, participating in the formation of the pre-metastatic niche and the regulation of distant organs. (5) Therapeutic resistance: Small extracellular vesicles transmit molecules like HTRA1 and miR-204-5p, conferring chemo- or targeted therapy resistance to recipient tumor cells.

The biological functions of sEVs in breast cancer are complex and critical. They serve not only as a core mediator for communication between tumor cells and between tumors and their microenvironment, but also exert multifaceted regulatory roles in tumor progression. Therefore, comprehensively elucidating the biological functions of sEVs is a fundamental prerequisite for achieving precise blockade of their pro-cancer effects and synergistically harnessing their anti-cancer potential (Figure 1).

#### 3.1.1 Regulation of tumor growth and proliferation

sEVs secreted by breast cancer cells serve as key mediators driving malignant tumor progression, significantly enhancing the proliferation and migration capabilities of recipient cancer cells. Research indicates that bioactive molecules carried by sEVs—such as peroxidasin homolog (PXDN), miRNAs, and N1ICD—participate in crucial intracellular pro-proliferative signaling pathways (Khraibah et al., 2025; Elayapillai et al., 2025; González-King et al., 2022; Ma et al., 2022), thereby promoting tumor growth through multiple mechanisms. Specifically, the long non-coding RNA SNHG4 enriched in sEVs can upregulate the expression of the nuclear export protein XPO5 (Wang Z. W. et al., 2025). The translational control tumor protein (TCTP) interacts with binding proteins such as DDX3, acting as a key regulator of sEV secretion and its RNA content (e.g., miRNA) (Amson et al., 2024), transmitting pro-growth signals during genotoxic stress and spontaneous tumorigenesis.

Additional studies reveal that sEVs released by IL-35-stimulated breast cancer cells downregulate THBS1 and PTEN while upregulating RRAS, CALM1, and other genes, thereby activating the classical Ras/Raf/MEK/ERK signaling pathway. This pathway activation further induces cyclin expression, directly driving cell cycle progression and accelerating proliferation (Liu et al., 2022). This process is accompanied by enhanced angiogenesis, synergistically supporting tumor growth by improving nutrient supply (Liu et al., 2022). This demonstrates the high integration and efficiency of tumor signaling. By regulating their own biogenesis, content loading, and signaling reprogramming of recipient cells, sEVs form a positive feedback loop promoting tumor growth. Ultimately, they synergistically drive the core malignant phenotypes of autonomous cell proliferation and dependent angiogenesis, revealing the deep-seated mechanism by which sEVs systematically advance tumor progression.

Regarding metabolic reprogramming, sEVs can serve as carriers of functional metabolic enzymes, intervening in the energy and material metabolism of recipient cells. Evidence indicates that sEVs not only directly deliver ATP citrate lyase (ACLY), Sirtuin-1 (SIRT1), and Sirtuin-6 (SIRT6) (Minic et al., 2022). Furthermore, miR-7641 derived from CAFs can indirectly upregulate HIF-1α, thereby activating the glycolysis-pentose phosphate pathway metabolic network and supplying precursor substances for nucleotide synthesis (Liu et al., 2021). This dual metabolic intervention, both direct and indirect, collectively drives metabolic reprogramming in breast cancer cells, thereby supporting their rapid proliferation and tumor growth. Notably, sEV functions are not always pro-tumorigenic. The study revealed that osteocyte-derived sEVs can deliver miR-148a-3p, which suppresses the ERK2/c-Myc signaling axis, thereby downregulating the expression of key nucleotide synthesis enzymes and consequently arresting the cell cycle (Shupp et al., 2021). However, cancer cells may also evolve corresponding escape mechanisms. For instance, sEVs derived from metastatic breast cancer cells actively export the La protein and its bound tumor-suppressive microRNAs (e.g., miR-122) via a p62-mediated selective autophagy pathway. This process relieves the inhibition of pyruvate kinase PKM2, thereby maintaining glycolytic flux and the supply of nucleotide synthesis precursors (Ngo et al., 2026), potentially sustaining their high proliferative state. In summary, sEVs exhibit functional plasticity in tumor regulation, with their ultimate effects determined by the dynamic interplay between cell origin, cargo composition, and microenvironmental signals.

#### 3.1.2 Driving invasion and metastasis

Multiple studies confirm that sEVs serve as multi-layered, systemic drivers of breast cancer invasion and metastasis. Their core mechanism lies in acting as efficient carriers of biological information, transmitting oncogenic signals between cells to induce EMT and establish pre-metastatic niches. For instance, bioactive molecules carried by sEVs (e.g., miRNAs, transmembrane proteins, GM1 ganglioside) can induce EMT (Nazari et al., 2024; Wang et al., 2021; Durán-Jara et al., 2024), activate the Wnt/β-catenin pathway (Ma et al., 2022), and enhance cell migration capacity (Nazari et al., 2024). sEVs derived from TNBC directly modify low-invasive breast cancer cells, inducing reduced cellular rigidity, cytoskeletal rearrangement, localized adhesion, and nuclear/cytoplasmic morphological changes to enhance their invasive capacity (Senigagliesi et al., 2022). *Clostridium* perfringens-derived sEVs (Fn-EVs) directly drive tumor growth and metastasis via the TLR4 signaling pathway (Li et al., 2023).

More critically, sEVs can transmit intact signaling pathway components across cells—such as the non-canonical Notch signaling component N1ICD or the oncogenic receptor ROR1/2—directly activating Notch, RhoA, or causing TGF-β signaling hyperactivation in recipient cells. This bypasses traditional ligand-receptor constraints to mediate pro-invasive phenotypes (González-King et al., 2022; Irmer et al., 2023; Teixeira et al., 2023). The hypoxic microenvironment further amplifies this role of sEVs. sEVs derived from hypoxic breast cancer cells (SEVh) enhance cell migration and extracellular matrix degradation by enriching bioactive molecules like matrix metalloproteinases (MMPs) and integrins (Bianca et al., 2022; Yang et al., 2025). Notably, under hypoxic conditions, sEVs modulate the activity balance between MMP-2 and MMP-9 by downregulating HIF-1α protein expression. While the gene expression of MMP-2 is generally upregulated, its protein expression and activity are significantly enhanced in the hypoxic group. Conversely, despite a slight decrease in MMP-9 gene expression, its activity is substantially elevated due to the action of sEVs. This divergence may stem from differing activation pathways mediated by MMP-1. Furthermore, the inhibition of TIMP-1 by the HIF signaling pathway in a hypoxic environment may preferentially favor MMP-2 activation, thus driving extracellular matrix remodeling and initiating pre-metastatic niche formation (Bianca et al., 2022). We found that sEVs drive local invasion and distant metastasis by remodeling cellular architecture, reconfiguring signaling pathways, and responding to environmental cues.

As multifunctional signaling carriers, sEVs also drive angiogenesis. Studies reveal they utilize surface proteins (e.g., CD44) for targeted delivery to endothelial cells. Upon entry, their cargo (e.g., EPHA2, GAS6) perform dual functions: directly activating endothelial cell signaling pathways (e.g., VEGF-AMPK) and indirectly activating cancer-associated fibroblasts (CAFs) (Zhang et al., 2023; Ikari et al., 2024; Li J. et al., 2025). Through this targeted delivery coupled with dual-regulation mechanism, sEVs not only directly stimulate angiogenesis but also systematically remodel the surrounding microenvironment, thereby supporting tumor vascular network formation.

sEVs can also exert long-distance effects on distant organs, establishing pre-metastatic niches. For instance, by delivering DPP3 to activate the Rap1 signaling pathway in pulmonary endothelial cells, they remodel the pulmonary vascular niche to facilitate cancer cell colonization (Li X. et al., 2025). Finally, external factors can amplify the pro-metastatic functions of sEVs. Conditions like chemotherapy (e.g., doxorubicin) or obesity upregulate specific proteins (PTX3, ECM1, etc.) in sEVs, unexpectedly accelerating lung metastasis and tumor growth (Wills et al., 2021; Xu et al., 2024). Through multidimensional actions—inducing EMT, transmitting oncogenic signals, activating stromal cells, and constructing pre-metastatic niches—sEVs form a core network for breast cancer invasion and metastasis.

#### 3.1.3 Establishing the immune microenvironment

By carrying immunosuppressive molecules, sEVs can induce T cell apoptosis, upregulate immune checkpoint expression, and promote the polarization of macrophages and neutrophils toward a pro-tumor phenotype. This shapes the immunosuppressive microenvironment of breast cancer, facilitating tumor progression and immune evasion. Studies reveal that sEVs can directly suppress antitumor immune responses by carrying PD-L1, FasL, TRAIL, and other immunosuppressive ligands directly into CD8^+^ and CD4^+^ T cells to initiate apoptosis. Alternatively, they activate the NF-κB pathway via enriched reticulin 4 (RTN4) to upregulate PD-L1, thereby inhibiting CD8^+^ T cell infiltration (Mondal et al., 2023; Wang H. et al., 2025).

Additionally, sEVs can polarize immune cells toward a pro-tumor phenotype, indirectly undermining the tumor-suppressive microenvironment. For instance, sEVs derived from triple-negative breast cancer induce regulatory T cell expansion by binding to the CD45 receptor via the Gal3BP-Gal3 complex, while simultaneously recruiting CAV1 protein to polarize neutrophils and promote lung metastasis (Raiter et al., 2023; Lin et al., 2024). TNF-α-regulated macrophage-derived sEVs also exhibit this function (Rodriguez-Baili et al., 2025). Further evidence supports sEVs' capacity for holistic remodeling of the immune microenvironment ecosystem. Brain-organ-derived sEVs (BO-sEVs) can upregulate PD-L1 expression in breast cancer cells, enhance cytokine secretion (including MCP-1, IL-6, IL-8) (Nazari et al., 2024), and further recruit and polarize immunosuppressive cells, collectively sustaining an immunosuppressive microenvironment.

#### 3.1.4 Mediating treatment resistance

Treatment resistance has become a major obstacle in breast cancer therapy (He et al., 2026). Studies indicate that sEVs released by TNF-α-regulated macrophages can induce and sustain the growth and proliferation of tumor stem cell-like subpopulations (CD44High/CD24Low phenotype) exhibiting resistance characteristics (Rodriguez-Baili et al., 2025). Tumor-derived sEVs also function as resistance vectors, delivering multiple functional molecules—including heat shock protein 70 and TGF-β1—to sensitive cells. Through synergistic effects such as enhancing glycolysis, inhibiting apoptosis, and boosting cell migration, they facilitate the lateral transmission of resistance phenotypes between cells (Hu et al., 2021; Tan C. et al., 2021). Thus, through multiple complementary pathways—shaping drug-resistant subpopulations, metabolic reprogramming, and transmitting survival signals—sEVs constitute a pivotal hub in the breast cancer resistance network, offering critical intervention targets for overcoming therapeutic resistance.

#### 3.1.5 Inducing systemic pathological effects

Small extracellular vesicles (sEVs) derived from breast cancer can mediate systemic pathological effects, influencing the physiological state of distant organs and tissues, thereby promoting tumor progression and related complications. Within the skeletal system, HTRA1 carried by sEVs upregulates MMP-13 expression in bone marrow osteoblast precursors, triggering proliferation of bone precursor cells, abnormal localization of hematopoietic stem cells, and accumulation of CD41^−^granulocyte-monocyte precursors within the bone marrow cavity—collectively disrupting skeletal homeostasis (Hao et al., 2023; Di et al., 2025; Cui et al., 2022). Furthermore, sEVs can deliver miR-204-5p to white adipose tissue, suppressing von Hippel-Lindau (VHL) gene expression. This stabilizes and upregulates hypoxia-inducible factor 1A (HIF1A) protein, activating the leptin signaling pathway (Hu et al., 2023). Ultimately, this promotes lipolysis and white adipose tissue browning, driving cancer-associated cachexia (Fei et al., 2021).

### 3.2 Communication networks of sEVs in breast cancer

Through understanding the biological functions of sEVs, we have discovered that in breast cancer, sEVs form a highly sophisticated and dynamic regulatory network via signal convergence and feedback loops, leveraging the specificity of their cargo and cellular origin. The functions and effects of this network are ultimately profoundly influenced by specific molecular mechanisms and breast cancer heterogeneity (Gummadi et al., 2025). Decoding the composition and operational logic of this network facilitates precise intervention to disrupt the malignant progression of breast cancer.

#### 3.2.1 Cargo-specificity and cellular origin specificity of sEVs

We discovered that the multifunctional nature of sEVs stems from the strict specificity of cargo loading and its close association with cellular origin. sEVs are not secreted homogeneously but represent signals actively packaged by specific cells under particular physiological or stress conditions. For instance, highly invasive triple-negative breast cancer cells release sEVs enriched with molecules such as mucin (Durán-Jara et al., 2024) and N1ICD (González-King et al., 2022), transmitting a pro-metastatic phenotype to neighboring cells. Conversely, osteoblasts in the bone microenvironment secrete sEVs carrying miR-148a-3p, exerting tumor-suppressive effects by inhibiting ERK2 signaling (Shupp et al., 2021), revealing the presence of antagonistic signals within the microenvironment. This specificity also manifests in targeting distant organs: sEVs targeting the lungs carry cargo dependent on CAV1 or ISG-modified DPP3 (Li X. et al., 2025; Lin et al., 2024), while those targeting the brain enrich for integrin β3 (Yang et al., 2025), both contributing to pre-metastatic niche formation.

#### 3.2.2 Signal convergence and feedback loops in sEVs

**FIGURE 2:**
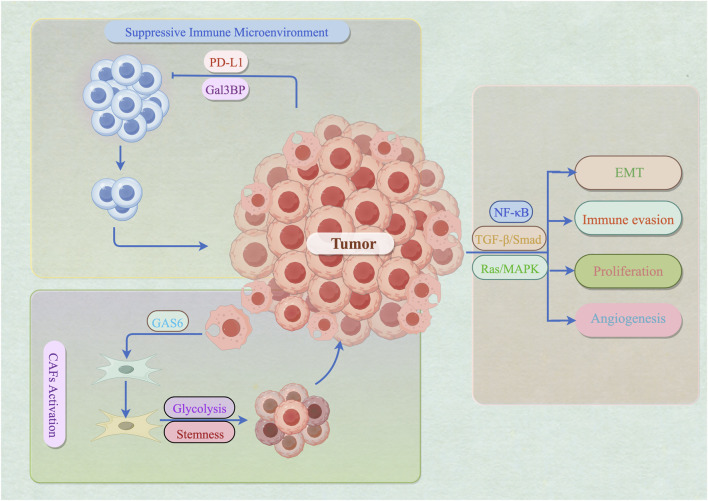
Positive Feedback Loops and Signal Convergence of sEVs in Breast Cancer (By Figdraw). Breast cancer cells secrete programmed death-ligand 1 (PD-L1) and Galectin-3-binding protein (Gal3BP), which suppress T cell cytotoxicity and induce T cell exhaustion, thereby establishing an immunosuppressive tumor microenvironment. Tumor-derived Gas6 (Growth Arrest-Specific Protein 6) activates adjacent fibroblasts, transdifferentiating them into cancer-associated fibroblasts (CAFs). Activated CAFs undergo metabolic reprogramming, fueling tumor growth through enhanced glycolysis, while acquiring enhanced stem-like properties, further supporting tumor progression and therapeutic resistance. Their signaling pathways include: (1) The common downstream signaling pathway NF-κB (Nuclear Factor kappa-light-chain-enhancer of activated B cells), promoting immune escape and inflammatory responses. (2) The TGF-β/Smad (Transforming Growth Factor-beta/Small Mothers against Decapentaplegic) pathway, inducing epithelial-mesenchymal transition and driving tumor metastasis. (3) The Ras/MAPK (Rat sarcoma/Mitogen-Activated Protein Kinase) pathway, promoting cell proliferation and angiogenesis, providing nutritional support for growing tumors.

Further analysis reveals that these cargo-specific sEVs do not transmit isolated signals but instead form dynamic, self-amplifying signaling networks between tumors and their microenvironments. The core mechanism involves establishing multiple positive feedback loops that converge on key oncogenic pathways. On the one hand, sEVs establish malignant positive feedback: tumor cells activate CAFs by delivering GAS6 protein via sEVs, while CAFs in turn enhance tumor cell stemness and glycolytic capacity through their own sEVs (Liu et al., 2021; Li J. et al., 2025). Simultaneously, tumor-derived sEVs deliver molecules like PD-L1 and Gal3BP to systemically suppress T-cell function and induce the release of immunosuppressive cells, creating a microenvironment conducive to tumor progression. This environment, in turn, promotes the release of even more immunosuppressive sEVs (Mondal et al., 2023; Raiter et al., 2023). On the other hand, despite the diverse cargo upstream, the effects of sEVs ultimately converge on several core signaling pathways—such as Ras/MAPK, TGF-β/Smad, and NF-κB (Elayapillai et al., 2025; Wang H. et al., 2025; Lin et al., 2024)—creating an amplifying effect that powerfully drives tumor proliferation, epithelial-mesenchymal transition, angiogenesis, and immune evasion (Figure 2).

#### 3.2.3 Dependency and heterogeneity

As previously described, the establishment and operation of this robust communication network exhibit strict molecular dependencies and are profoundly influenced by the inherent heterogeneity of breast cancer itself. This constitutes its immense complexity and suggests both potential targets and challenges for intervention. Each step of sEVs—from biogenesis, cargo sorting, and release to recognition and uptake by target cells—relies on key molecules such as Rab27A, B3GALT4, p62/La, ARF4, and CD44 (Elayapillai et al., 2025; Ma et al., 2022; Ngo et al., 2026; Ikari et al., 2024; Li X. et al., 2025). Disrupting these pathways can dismantle specific communication chains. Simultaneously, sEV function exhibits profound context dependence, with the same molecule (e.g., miR-205) potentially playing diametrically opposed roles across different breast cancer subtypes or disease stages (Elayapillai et al., 2025). sEVs derived from triple-negative breast cancer often exhibit stronger pro-metastatic and immunosuppressive properties. This heterogeneity implies that diagnostic or therapeutic strategies targeting sEVs must be predicated on precise identification of their cellular origin, physiological context, and core cargo. Understanding this sEV communication system—defined by specificity, interconnectedness, and complexity—not only offers novel insights into breast cancer progression mechanisms but also paves the way for developing novel liquid biopsy-based biomarkers and precision therapies targeting this signaling network.

### 3.3 Translational potential of sEVs in breast cancer

sEV-related technologies are transitioning from basic research to clinical translation, with liquid biopsy and carrier-based therapy studies being particularly prominent. This is poised to become a core driver in revolutionizing breast cancer diagnosis and treatment, playing a crucial role in overcoming key clinical bottlenecks such as early diagnosis, overcoming drug resistance, and improving prognosis.

#### 3.3.1 Biomarkers for liquid biopsy

As a cutting-edge liquid biopsy tool, sEVs carry rich molecular information that opens new pathways for precision diagnosis and treatment of breast cancer. Studies reveal significantly elevated expression of epithelial cell adhesion molecule (EpCAM) and the specific presence of enolase 1 (ENOL1) in sEVs, offering highly promising tools for early breast cancer detection (Ngo et al., 2025; Salmond et al., 2025). Concurrently, the discovery of specific lipid molecules LysoPC 22:6/0:0 and N-acetyl-L-phenylalanine in the MDA-MB-231 cell model (D'Mello et al., 2024) suggests the potential of metabolites as novel early-stage breast cancer diagnostic markers.

The value of sEVs as biomarkers is particularly prominent in the precise molecular typing and subtype identification of breast cancer. For TNBC, the high-specificity enzymatic activity of aldolase (ALDOA), phosphoglycerate kinase 1 (PGK1), and enolase (ENO) in its sEVs, along with the unique presence of peroxidasin homolog (PXDN), glutathione hydrolase 5 proenzyme (GGT5), and plasminogen activator inhibitor 1 (SERPINE1) constitute a distinctive fingerprint profile distinguishing it from other subtypes (Khraibah et al., 2025; Minic et al., 2023). For inflammatory breast cancer (IBC), the miRNA signature profile derived from sEVs (upregulation of miR-181b-5p and miR-222-3p, downregulation of let-7a-5p) demonstrates superior diagnostic efficacy, with the combination of let-7a-5p and miR-222-3p further enhancing diagnostic accuracy (Ahmed et al., 2021). Transmembrane serine protease ST14 and claudin-3 (CLDN3) have been proposed as EV biomarkers with broad-spectrum potential (Hüttmann et al., 2024).

In therapeutic response prediction and prognostic assessment, sEVs demonstrate unique advantages for dynamic monitoring. Studies indicate that combined detection of sEV-derived annexin A2 (AnxA2) protein and mRNA holds promise as a key indicator for predicting chemotherapy responsiveness in aggressive TNBC patients, providing a basis for personalized treatment (Desai et al., 2022). Thus, sEV-derived biomarkers span diverse levels—from metabolites and functional proteins to non-coding RNAs—demonstrating significant potential in breast cancer early detection, molecular subtyping, and treatment prognosis prediction.

#### 3.3.2 As therapeutic vectors or targets

In recent years, sEVs have emerged as a hotspot across research fields for their role as therapeutic vectors or targets in breast cancer. Research indicates that by loading drugs or bioactive molecules, sEVs can overcome limitations of conventional therapies. For instance, co-loading the photosensitizer Ce6 with the neutral sphingolipase (nSMase) inhibitor GW4869 into sEVs derived from bone marrow mesenchymal stem cells enables the construction of immunomodulatory photosensitive nanovesicles (Ding et al., 2023). Dendritic cell-derived sEVs loaded with poorly water-soluble berberine hydrochloride enhanced stability and delivery efficiency, inhibiting breast cancer proliferation and migration (Salek et al., 2022). For nucleic acid delivery, strategies such as cationic lipid modification, chiral graphene quantum dot enhancement, or pH-responsive peptide assistance enable sEVs to efficiently deliver nucleic acid therapeutics like siRNA and miR-34a, achieving gene silencing or replacement therapy to overcome chemotherapy resistance (Klyachko et al., 2024; Kim G. et al., 2025). Furthermore, engineered modifications can endow sEVs with active targeting capabilities, such as displaying HER2 antibodies, conjugating AS1411 aptamers, or VEGFR-targeting peptides (Xing et al., 2023; Hong et al., 2025; Kim Y. M. et al., 2025), significantly enhancing their specific recognition and enrichment toward breast cancer cells. Further studies have established a paromomycin-induced protein interaction system enabling controlled cargo loading onto sEVs, providing a novel tool for precision delivery (Kim and Kim, 2023).

As discussed, tumor-derived sEVs serve as critical mediators of immune evasion and pre-metastatic niche formation. Thus, inhibiting their secretion or neutralizing their activity represents a key therapeutic strategy. Drugs such as GW4869 and eribulin can inhibit sEV release, thereby improving the immune microenvironment and reducing metastatic propensity (Kim G. et al., 2025; Pederson et al., 2021; Giulietti et al., 2024). Meanwhile, temsirolimus activates autophagy to specifically inhibit PD-L1 secretion on sEV surfaces, synergizing with immune checkpoint blockade therapy (Park et al., 2022). For direct clearance, DNA network-based sEV trap (DNET) can specifically capture and photodynamically destroy tumor sEVs, disrupting their pro-cancer signaling (Yang et al., 2024). Additionally, regulating sEV content secretion is a key approach: losartan blocks metastasis-promoting functions by inhibiting DPP3 modification and vesicular secretion (Li X. et al., 2025); while the marine alkaloid Manzamine A alters sEV composition and inhibits breast cancer progression by regulating RIP1-mediated secretory autophagy (Wang et al., 2023).

sEVs demonstrate significant potential in activating anti-tumor immunity. For instance, HER2-targeted vesicles loaded with GSDMD-N mRNA induce pyroptosis and stimulate immune responses within tumors (Xing et al., 2023); Dendritic cell-derived sEVs expressing membrane-bound IL-2 and co-stimulatory molecules can substitute antigen-presenting cell functions, activate T cells, and enhance immunotherapy efficacy. Combining them with natural compounds (e.g., macbecin II) or chemotherapeutic agents further create synergistic effects, promoting immune cell infiltration and inhibiting tumor growth and metastasis (Wu et al., 2023; Deshpande et al., 2025). As both engineerable smart delivery vehicles and key targets driving tumor progression, breast cancer treatment approaches can be achieved by loading sEVs with drugs, nucleic acids, or immunomodulatory factors supplemented with targeted modifications, or by inhibiting their secretion, clearing circulating sEVs, and regulating their biological contents to reverse sEV-mediated immune suppression and metastasis processes. The deep integration and combined application of these strategies offer novel, precise, and translationally promising solutions for breast cancer treatment, particularly for triple-negative breast cancer (Table 1).

**TABLE 1:** Breast Cancer Treatment Strategies Targeting *sEVs*.

Category	Core strategies	Key molecules/Methods	Functions	Citation
As a delivery vehicle	Drug/Nucleic acid delivery	Ce6, berberine hydrochloride, siRNA/miRNA	Targeted therapy to overcome drug resistance	Klyachko et al. (2024), Kim G. et al. (2025)
	Targeted engineering	HER2 antibody, AS1411 aptamer	Precise identification and enhanced enrichment	Xing et al. (2023), Hong et al. (2025), Kim Y. M. et al. (2025)
	Controlled loading	Pareomycin induction system	Achieve on-demand loading	Kim and Kim (2023)
As an intervention target	Secretion inhibition	GW4869, eliplin, tacrolimus	Achieve on-demand loading	Kim G. et al. (2025), Pederson et al. (2021), Giulietti et al. (2024), Park et al. (2022)
	Direct clearance	DNA network-based sEV trap (DNET)	Block pro-cancer signaling pathways	Yang et al. (2024)
	Content regulation	Losartan, manzamine A	Alter function and inhibit metastasis	Li X. et al. (2025), Wang et al. (2023)
For immune activation	Immune molecule loading	GSDMD-N mRNA	Induce pyroptosis to activate immunity	Xing et al. (2023)
	Engineered immunovesicles	Membrane-bound IL-2, co-stimulatory molecules	Replace antigen-presenting cells to activate T cells	Xing et al. (2023)
	Combination therapy	Macbecin II, chemotherapy drugs	Synergistic effects to suppress tumors	Wu et al. (2023), Deshpande et al. (2025)

## 4 Discussion

This review systematically examines the biological functions of sEVs throughout the entire course of breast cancer development, extending beyond previous focuses on immunotherapy and diagnostics (Zhou et al., 2024; Galardi et al., 2025). We elucidate mechanisms by which cellular origins regulate the cargo specificity of small extracellular vesicles and summarize recent advances in their application as biomarkers and therapeutic agents. Crucially, we propose that small extracellular vesicles constitute a robust, heterogeneously regulated communication system that sustains malignant progression and therapeutic resistance by orchestrating signaling cascades, feedback loops, and immune reprogramming.

### 4.1 sEVs as core drivers of systemic regulation in breast cancer

sEVs in breast cancer have transcended their role as mere signaling molecules, now acting as system-level regulatory hubs that integrate and drive the entirety of tumorigenesis, progression, immune evasion, therapeutic resistance, and systemic pathological alterations. This is achieved by constructing a highly dynamic, self-reinforcing intercellular communication network that links tumor cells, stromal cells within the microenvironment, and distant organs into a functional continuum, systematically advancing the malignant evolution of breast cancer. Specifically, sEVs promote the proliferative, invasive, and metastatic capabilities of tumor cells through multiple dimensions by delivering a diverse array of bioactive cargoes, including proteins, nucleic acids, and metabolic enzymes. For instance, they drive proliferation by activating core signaling pathways such as Ras/MAPK (Elayapillai et al., 2025) or promote dissemination by inducing epithelial-mesenchymal transition (EMT) and establishing pre-metastatic niches (Li X. et al., 2025). Concurrently, sEVs act as remodelers of the tumor microenvironment by delivering immunosuppressive signals (e.g., PD-L1) to polarize immune cells (Raiter et al., 2023), inhibit T cell function (Mondal et al., 2023), and activate stromal cells like cancer-associated fibroblasts (CAFs) (Li J. et al., 2025), collectively fostering a local milieu conducive to tumor growth and immune evasion. In the context of therapy, sEVs serve as vectors for drug-resistant phenotypes, horizontally transferring resistance signals between cells (Hu et al., 2021; Tan C. et al., 2021) and maintaining tumor-initiating cell populations (Rodriguez-Baili et al., 2025), thereby mediating therapeutic resistance. Furthermore, sEVs derived from breast cancer can overcome local confinement, exerting long-range effects via the circulatory system to disrupt bone homeostasis (Cui et al., 2022) and induce cachexia (Fei et al., 2021), among other systemic pathological consequences. This broad and profound regulatory capacity stems from the specificity of their cargo loading and cellular origin, leading to the formation of multi-layered signaling convergence and positive feedback loops.

### 4.2 sEVs and breast cancer heterogeneity

The biogenesis and functional execution of sEVs exhibit strict cell-of-origin specificity and molecular sorting specificity, serving as a pivotal hub for decoding breast cancer heterogeneity. This specificity is not a passive response but stems from active cellular programming. Specifically, the type and state of the secreting cell determine sEV function. Breast cancer cells of different molecular subtypes (e.g., ER+, HER2+, TNBC) secrete sEVs carrying distinct cargo molecules due to inherent differences in signaling pathway activity and expression profiles. For instance, highly invasive TNBC cells tend to sort sEVs enriched with mucin (Durán-Jara et al., 2024), integrins (Yang et al., 2025), and specific oncogenic non-coding RNAs (e.g., SNHG4) (Wang Z. W. et al., 2025), thereby driving EMT and organ-targeted metastasis. Conversely, sEVs derived from HER2-overexpressing breast cancers may exhibit distinct pro-growth signaling profiles (Hao et al., 2021). Furthermore, the cargo composition of sEVs undergoes dynamic reprogramming within the same tumor among different cell subpopulations or among homologous cells exposed to microenvironmental stimuli such as hypoxia, nutrient deprivation, or drug treatment (Bianca et al., 2022; Wills et al., 2021), thereby outputting adaptive pro-survival, pro-metastatic, or immunosuppressive signals.

Beneath cellular origin differences, the molecular mechanisms of cargo sorting are the executors of specific signaling and functional heterogeneity. The loading of sEV contents is a highly selective and tightly regulated process dependent on complex molecular mechanisms. These include membrane sorting mediated by the ESCRT complex, transmembrane protein networks, and lipid raft microdomains (Wang et al., 2021; Sharma et al., 2025), alongside nucleic acid sorting facilitated by specific RNA-binding proteins such as hnRNPA2B1 and Y-box protein 1 (Li X. et al., 2025; Liu Z. et al., 2024). This process is further coordinated by an integrated regulatory hub comprising upstream signaling pathways (e.g., TGF-β, HIF-1α, NF-κB (Wang H. et al., 2025; Lin et al., 2024)) and key regulatory proteins (e.g., Rab27 A/B, ARF4 (Elayapillai et al., 2025)), collectively determining sEV biogenesis, release, and cargo profiles. Thus, breast cancer heterogeneity—encompassing molecular subtypes, spatial heterogeneity, and therapeutic response variability—is precisely mapped onto the molecular characteristics of distinct sEV subpopulations. The functional heterogeneity of sEVs essentially represents a functional mirroring and systemic amplification of their source cells' molecular features and adaptive states. This provides an integrated explanatory framework based on sEV regulatory networks to resolve observational discrepancies among studies in this field.

### 4.3 Tumor-immune ecosystem remodeling

sEVs serve as a core mediator in reshaping the tumor-immune ecosystem, driving therapeutic resistance, and maintaining tumor malignant phenotype memory. Their role spans the entire progression of breast cancer, establishing the biological foundation for their emergence as novel biomarkers and therapeutic targets. Through systematic intercellular communication, sEVs construct a locally immunosuppressive tumor microenvironment. As biomarkers, the cargo carried by sEVs—such as PD-L1, specific miRNA profiles, and phosphorylated proteins—directly reflects the tumor’s real-time immune evasion status, activated resistance signaling, and molecular subtype (Mondal et al., 2023; Minic et al., 2023). This makes sEVs an ideal liquid biopsy tool for dynamically monitoring treatment efficacy, predicting resistance, and assessing prognosis. As therapeutic targets, inhibitors (e.g., GW4869) can reduce tumor-derived sEV secretion (Ding et al., 2023), or traps can be developed to clear circulating harmful sEVs (Minic et al., 2023; Yang et al., 2024) to disrupt their pro-cancer communication networks. Alternatively, sEVs can be engineered to carry chemotherapeutic agents (Yang et al., 2024), nucleic acid therapeutics (e.g., siRNA, miRNA) (Xing et al., 2023), or immune agonists (Guo et al., 2025), leveraging their inherent delivery capacity and targeting properties to transform them into precision weapons capable of striking tumors, reversing resistance, or activating anti-tumor immunity.

### 4.4 The “causal-correlated” association system between sEVs and breast cancer

We discovered that the relationship between sEVs and breast cancer is not merely a simple correlation, but rather a multi-layered, dynamic causal association. sEVs are not only passive reflections of breast cancer’s existence but also key effectors actively driving its malignant progression. Numerous functional experiments provide direct evidence for this causal relationship. For instance, co-culturing breast cancer cells with sEVs secreted by these cells alongside normal or low-invasiveness cells, or directly injecting such sEVs into model animals, leads to observable effects in recipient cells: enhanced proliferation, invasion, EMT, drug resistance, and the formation of pre-ecological niches for distant organ metastasis (Li X. et al., 2025). Conversely, specific inhibition of sEV secretion or key cargo loading via genetic or pharmacological means (e.g., Rab27a knockdown, GW4869 treatment) significantly blocks these pro-cancer phenotypes (Elayapillai et al., 2025; Ding et al., 2023). This indicates that specific molecular cargo carried by sEVs constitutes a sufficient cause for inducing malignant transformation in recipient cells. As biomarkers associated with breast cancer, the molecular profiles of sEVs (e.g., specific proteins, miRNAs, lipids) show strong correlations with breast cancer molecular subtypes, disease staging, treatment response, and prognosis. For instance, elevated levels of PD-L1 or specific integrins in plasma sEVs from triple-negative breast cancer patients correlate with poorer prognosis and resistance to immunotherapy (Yang et al., 2025; Lin et al., 2024). This association provides a foundation for non-invasive liquid biopsy but does not directly prove that sEVs cause these clinical outcomes. Thus, the relationship between breast cancer and sEVs represents a “causal-correlated” unity.

### 4.5 Limitations and future prospects

Despite this systematic review of sEVs' multifaceted roles in breast cancer, several limitations remain. (1) While multiple evidence categories were reviewed, their varying levels of evidence were not systematically differentiated. Although functional experiments support the critical role of sEVs in breast cancer and their potential as core mechanisms and therapeutic targets in specific molecular pathways in humans, validation remains contingent upon further clinical-relevant models (e.g., human organoids, PDX models) and prospective clinical studies. (2) Research on sEVs as diagnostic biomarkers and therapeutic carriers/targets remains predominantly preclinical, with most evidence derived from *in vitro* experiments and animal models. Clinical translation is still in its early stages and requires substantial clinical evidence to support it. Future successful translation hinges on overcoming critical bottlenecks, including scalable production, quality control, targeted delivery, and validation of clinical benefits. (3) Constrained by current technology and the completeness of the evidence chain, this paper’s exploration of tumor-suppressive sEVs is limited, potentially underestimating the complexity of the dynamic equilibrium network among sEVs of different origins *in vivo*.

To address the aforementioned limitations, corresponding strategies and pathways can be adopted for improvement. Regarding the level of evidence, future research can refer to the evidence grading systems of systematic reviews and evidence-based medicine. When sorting and integrating various studies (including cell experiments, animal models, and clinical observations), the sources and levels of evidence should be systematically labeled, and research results from clinical studies and high-level preclinical models should be prioritized to enhance the credibility and clinical guidance value of conclusions. In promoting clinical translation, a collaborative innovation platform integrating industry, academia, research, and medicine can be built to jointly overcome key technical bottlenecks such as large-scale production of sEVs, quality standardization, and optimization of targeted delivery systems. Based on this, standardized prospective clinical trials can be designed to comprehensively evaluate their accuracy as diagnostic biomarkers, safety as therapeutic carriers, and clinical benefits. To further reveal the complex network of sEVs' roles in the tumor microenvironment, high-resolution technologies such as single vesicle analysis, *in vivo* imaging, and spatial multi-omics should be continuously developed. Furthermore, more clinically relevant research systems such as organoids and humanized animal models should be comprehensively utilized to deeply elucidate the dynamic balance and regulatory mechanisms of tumor-promoting and tumor-suppressing sEVs, thereby gaining a more systematic and comprehensive understanding of the dual role of sEVs in breast cancer progression.

Future research requires moving beyond linear causal thinking to adopt a systemic, dynamic analytical perspective for understanding sEV functions in breast cancer. At the translational level, patient-specific sEV analysis could establish a closed-loop diagnostic-therapeutic system encompassing “monitoring-diagnosis-treatment-reassessment”. This approach would identify primary pathogenic pathways through dynamic monitoring, design personalized engineered sEV interventions, and adjust strategies in real-time based on sEV feedback. Realizing this vision requires prioritizing three foundational tasks: (1) establishing more rigorous standards for sEV isolation and identification; (2) validating mechanisms in physiologically relevant models such as organ-on-a-chip and genetically engineered animals; (3) integrating systems biology tools like single-vesicle analysis and spatiotemporal omics to comprehensively decipher the dynamic heterogeneity of sEVs.

## 5 Conclusion

This study systematically elucidates the multifaceted biological functions of sEVs in breast cancer, reveals their intrinsic mechanisms as key intercellular communication mediators driving tumor progression, and explores their significant translational value in liquid biopsy and targeted therapy. Findings indicate that sEVs serve as core drivers and information hubs in breast cancer progression. By delivering specific contents, they establish a dynamically regulated network that promotes tumor growth, metastasis, immune evasion, and drug resistance development. Notably, sEV functions exhibit high context dependency and bidirectional regulatory potential, providing both a critical molecular basis for targeted breast cancer interventions and positioning them as highly promising liquid biopsy biomarkers and engineered therapeutic carriers. Currently, sEV-related research has progressively evolved into a complete closed-loop system spanning fundamental mechanism elucidation to clinical application development. This not only deepens theoretical understanding of breast cancer heterogeneity and metastatic mechanisms but also offers innovative perspectives and feasible pathways for overcoming current diagnostic and therapeutic bottlenecks while advancing personalized precision medicine.
